# First person – Karishma Chhabria

**DOI:** 10.1242/dmm.042085

**Published:** 2019-09-01

## Abstract

First Person is a series of interviews with the first authors of a selection of papers published in Disease Models & Mechanisms (DMM), helping early-career researchers promote themselves alongside their papers. Karishma Chhabria is first author on ‘Sodium nitroprusside prevents the detrimental effects of glucose on the neurovascular unit and behaviour in zebrafish’, published in DMM. Karishma conducted the research described in this article while a PhD student in Tim Chico and Clare Howarth's lab at University of Sheffield, UK. She is now a postdoc in the lab of David Kleinfeld at UCSD, USA, investigating how the brain achieves precise spatio-temporal blood-flow regulation.


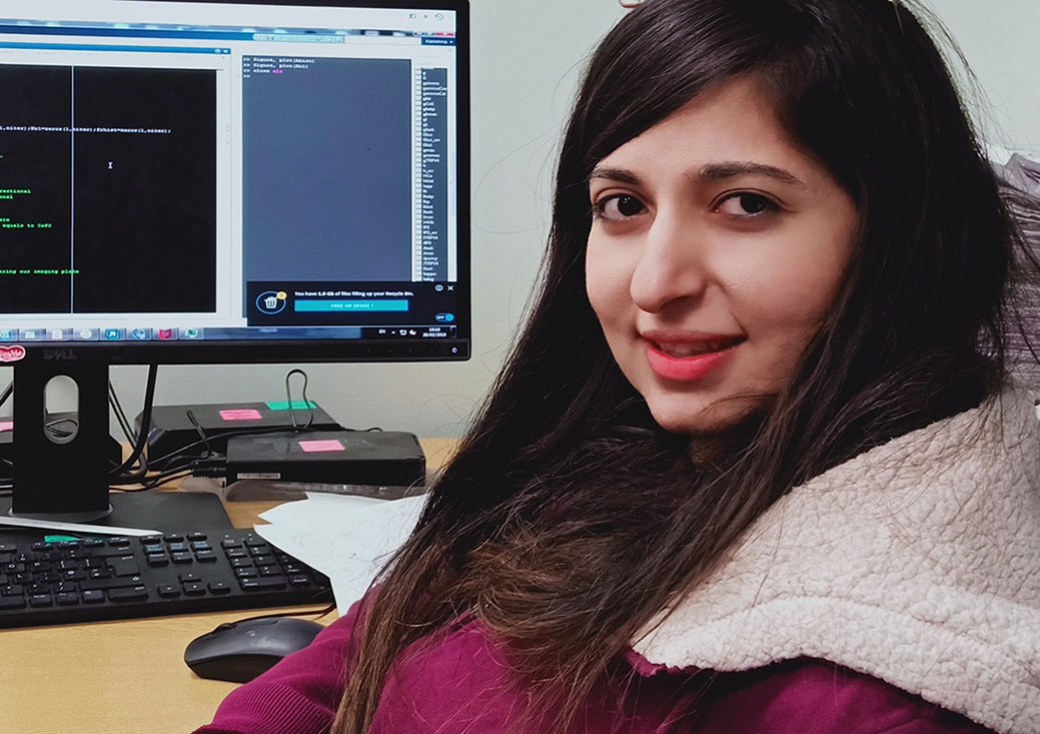


**Karishma Chhabria**

**How would you explain the main findings of your paper to non-scientific family and friends?**

Diabetes affects around 1 in 11 of the world's adult population and poses a risk for both heart and brain diseases. A common feature of type 2 diabetes is high blood-glucose levels. In our study, we investigate the effect of feeding high glucose to zebrafish on brain function and behavior. We find that high-glucose-fed zebrafish have several abnormities in various cells in the brain and also display abnormal behavior. Intriguingly, we observed that all the negative effects of high-glucose exposure on the brain and behavior can be treated with a drug that artificially increases nitric oxide in the brain. It has commonly been shown that there is a deficiency of nitric oxide in the brain under diabetes; however, there are no existing nitric-oxide-based therapeutic treatments for diabetes. Our study shows for the first time that nitric-oxide donors could be used as potential therapeutics to treat diabetes and related brain dysfunctions.

“Our study shows for the first time that nitric-oxide donors could be used as potential therapeutics to treat diabetes and related brain dysfunctions.”

**What are the potential implications of these results for your field of research?**

The human brain is energetically expensive, consuming around 20% of the cardiac output while accounting for only 2% of the body weight. Neurons don't have their own energy reserves and therefore rely on a continuous supply of blood flow to receive the oxygen and glucose needed for their survival. The matching of this energy supply and demand is achieved through a phenomenon called neurovascular coupling. There has been a recent increase in research investigating the importance of neurovascular coupling in disease; however, the precise mechanisms are not known. In our earlier paper, we showed that high-glucose exposure impairs neurovascular coupling and in the current study we have shown dysfunction of the critical cells known to mediate neurovascular coupling. Our current study applies multiscale research methodologies, showing high-glucose-induced dysfunctions from the cellular level through to behavior.

**What are the main advantages and drawbacks of the model system you have used as it relates to the disease you are investigating?**

The biggest advantage of using larval zebrafish as a model system for investigating disease is the ease of screening several prospective drugs for various diseases. Larval zebrafish at 8 days post-fertilization are capable of performing complex visual tasks like object preference, thus presenting the ability to also investigate mechanisms of behavioral dysfunctions. Additional advantages are the ease of manipulating genes and the optical transparency of the larval forms.

In our current study, we have investigated the effect of high-glucose exposure, a pathology of diabetes. However, to understand the disease in its entirety, development of a larval zebrafish model of diabetes would be ideal. Although streptozotocin-induced diabetes models exist, these are more commonly available in adult zebrafish. The ability to image neurovascular function in adult zebrafish using state-of-the-art lightsheet microscopy would be a step forward, allowing us to more fully investigate the pathological consequences of diseases.

“The biggest advantage of using larval zebrafish as a model system for investigating disease is the ease of screening several prospective drugs for various diseases.”

**What has surprised you the most while conducting your research?**

There are two main aspects of my research that I found particularly striking, including our observations of neuronal hyperexcitability and TRPV4 underexpression induced by glucose exposure. Secondly, I was surprised by the large effect of the nitric-oxide donor in reversing all the detrimental effects of the high-glucose exposure. This could have immense potential for investigating novel nitric-oxide-based therapeutics to treat diabetes and related brain dysfunction.

**Figure:**
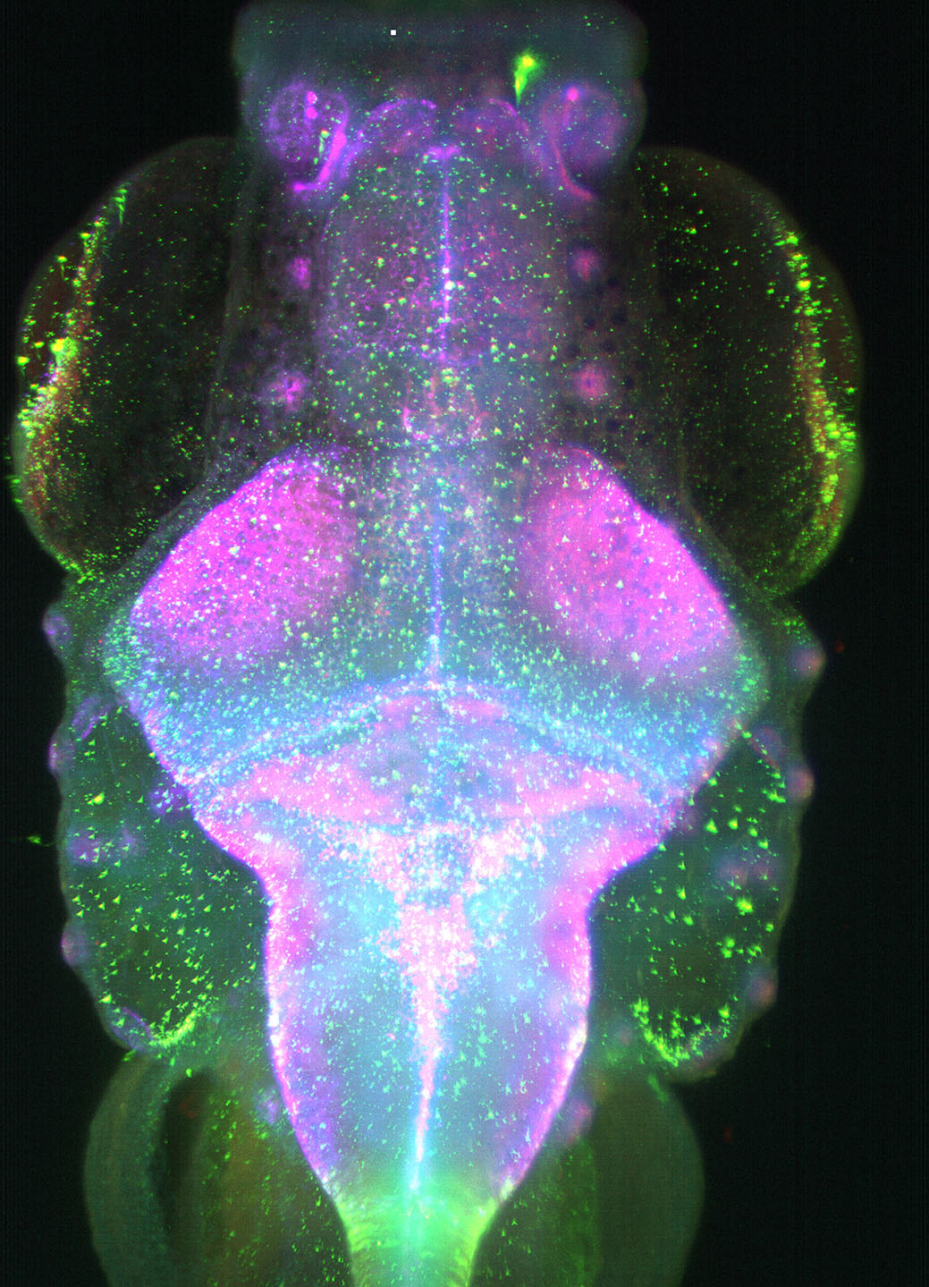
**Dorsal view of a 9-day-old zebrafish brain stained for different cellular markers (green: glutamate receptors on neurons and glial cells, red: phalloidin stain for neuronal axons and radial glial processes, cyan: DAPI stain for nuclear localization, and blue: glutamine synthase in the radial glial cells).**

“This could have immense potential for investigating novel nitric-oxide-based therapeutics to treat diabetes and related brain dysfunction.”

**Describe what you think is the most significant challenge impacting your research at this time and how will this be addressed over the next 10 years?**

There are several important challenges affecting neurovascular research and its implications in diseases. Studies have shown that some neurovascular abnormalities occur before the onset of neurodegenerative disorders. There is a need to study the progression of a disease longitudinally, to find the precise cause of the neurovascular dysfunction which may predispose the brain to neurodegeneration. Diabetes, proposed to be an important risk factor for neurodegenerative disease, causes cognitive impairments. It is essential to understand the mechanisms of diabetes to understand the basis of associated cognitive effects. Another challenge is the ability to visualize the dynamics of multiple cells *in vivo* in order to understand their interactions in real time. For instance, we want to be able to simultaneously image the responses of neurons, astrocytes, smooth muscle cells, pericytes, endothelial cells, microglia and blood flow to sensory stimulation in health and disease. Addressing such challenges will increase our understanding not only of the fundamental workings of the brain but also what goes wrong in disease.

**What's next for you?**

I am currently examining the fundamental mechanisms of cerebral blood flow regulation in Prof. Kleinfeld's laboratory, UCSD, as a postdoctoral researcher. My research aims to understand the basis of vasomotion, a spontaneous fluctuation in the blood flow that underlies resting-state fMRI and is impaired in various diseases such as Alzheimer's disease.

## References

[DMM042085C1] Chhabria K., Vouros A., Gray C., MacDonald R. B., Jiang Z., Wilkinson R. N., Plant K., Vasilaki E., Howarth C. and Chico T. J. A. (2019). Sodium nitroprusside prevents the detrimental effects of glucose on the neurovascular unit and behaviour in zebrafish. *Dis. Model. Mech.* 12, dmm039867 10.1242/dmm.039867 31481433 PMC6765192

